# Can Ileostomy Reversal Be Safely Performed by Surgical Residents?

**DOI:** 10.3390/medicina60111847

**Published:** 2024-11-09

**Authors:** Michał Kisielewski, Magdalena Pisarska-Adamczyk, Natalia Dowgiałło-Gornowicz, Łukasz Nawacki, Wojciech Serednicki, Mateusz Wierdak, Jerzy Wilczek, Kamil Safiejko, Marcin Juchimiuk, Marian Domurat, Jacek Pierko, Mateusz Mucha, Wojciech Fiedorowicz, Michał Wysocki, Maurycy Ladziński, Michał Zdrojewski, Tomasz Sachańbiński, Tomasz Wojewoda, Victoria Chochla, Karol Tkaczyński, Michał Jankowski, Wojciech M. Wysocki

**Affiliations:** 1 Chair of Surgery of the Faculty of Medicine and Health Sciences, Andrzej Frycz Modrzewski University, 30-705 Krakow, Poland; 2 Department of General and Oncological Surgery, 5th Military Clinical Hospital, 30-901 Krakow, Poland; 3 Department of Medical Education, Medical College, Jagiellonian University, 30-688 Krakow, Poland; 4 Department of General, Minimally Invasive and Elderly Surgery, University of Warmia and Mazury, 10-719 Olsztyn, Poland; 5 Collegium Medicum, Jan Kochanowski University, 25-317 Kielce, Poland; 6 2nd Department of General Surgery, Jagiellonian University, 30-688 Krakow, Poland; 7 Department of Oncological Surgery, Specialist Hospital, 36-200 Brzozow, Poland; 8 Colorectal Cancer Unit, Maria Skłodowska-Curie Białystok Oncology Center, 15-027 Białystok, Poland; 9 Department of General Surgery and Surgical Oncology, Ludwik Rydygier Memorial Hospital, 31-826 Krakow, Poland; 10 “Pro-Medica” Hospital, 19-300 Elk, Poland; 11 Oncological Surgery Clinic, MSWiA Hospital, 10-228 Olsztyn, Poland; 12 Oncological Surgery Department with a Sub-Department of Breast Diseases, Tadeusz Koszarowski Oncology Centre, 45-061 Opole, Poland; 13 Institute of Medical Sciences, Faculty of Medicine, University of Opole, 45-040 Opole, Poland; 14 Department of Oncological Surgery, 5th Military Hospital, 30-901 Krakow, Poland; 15 Department of Surgical Oncology, Oncology Center, Prof. Franciszek Łukaszczyk Memorial Hospital, 85-796 Bydgoszcz, Poland; 16 Chair of Surgical Oncology, Ludwik Rydygier’s Collegium Medicum in Bydgoszcz, Nicolaus Copernicus University, 87-100 Toruń, Poland; 17 National Institute of Oncology, Maria Skłodowska-Curie Memorial, 02-781 Warsaw, Poland

**Keywords:** colorectal surgery, ileostomy reversal, surgical training, surgical residents, postoperative complications, loop ileostomy

## Abstract

*Background and Objectives*: The growing number of colorectal cancer patients has highlighted the importance of surgical education in colorectal surgery. Despite the negative impact of the COVID-19 pandemic on surgical training, recent changes in the Polish surgical training program have increased the number of intestinal procedures required to be completed by residents. This study aims to assess the safety of ileostomy reversal procedures performed by surgical residents. *Materials and Methods:* A multicenter prospective cohort study, the LILEO study, was conducted from October 2022 until December 2023 across 20 Polish surgical departments. The study included 199 patients who underwent ileostomy reversal and were divided into two groups: 139 patients operated by specialist surgeons and 60 patients operated by surgical residents. The primary outcomes measured were postoperative complications, length of hospital stay (LOS), and 30-day reoperation rate. Secondary outcomes included the severity of perioperative complications assessed using the Clavien–Dindo classification and a focused analysis of loop ileostomy reversal outcomes. *Results:* The median LOS was significantly shorter in the resident group (5.5 days vs. 6 days, *p* < 0.05). Although the overall complication rate was lower in the resident group (21.7% vs. 33.1% in the specialist surgeon group), this difference was not statistically significant (*p* = 0.105). The 30-day reoperation rate was 3.3% in the resident group and 8.6% in the specialist surgeon group (*p* = 0.179). In terms of severity, minor complications (Clavien–Dindo grades 1 and 2) were more common in the specialist group (*p* < 0.05). The analysis of loop ileostomy reversals revealed no significant differences in postoperative outcomes between the two groups. *Conclusions:* Ileostomy reversal procedures performed by surgical residents under supervision are safe and feasible, with outcomes comparable to those performed only by specialist surgeons. These findings support ileostomy reversal as a valuable procedure for developing surgical residents’ skills and do not negatively affect postoperative outcomes.

## 1. Introduction

Surgical education in colorectal surgery is especially important due to the rapidly growing number of colorectal cancer patients. Currently, colorectal cancer ranks as the third most prevalent cancer globally [1]. During the COVID-19 pandemic, surgical education deteriorated due to the decreased number of colorectal procedures. Taking into consideration the aforementioned statistics, such a highly prevalent cancer must be diagnosed in a timely fashion and treated to improve patient outcomes and decrease socioeconomic burdens [2]. Changes in the Polish surgical training program led to an increase in the number of obligatory intestinal procedures conducted during the 6-year training period, from 65 procedures in 2018 to 146 procedures in 2023. Evidence showing that procedures performed by residents are safe and feasible is crucial in order to further develop colorectal surgery education in Poland.

Ileostomy reversal is a basic colorectal procedure that includes the anastomosis of two small bowel parts. Access to the bowel ends in the majority of loop ileostomy reversal cases is through the ileostomy site, but the end ileostomy reversal can require laparotomy and more extensive intra-abdominal preparation. Ileostomy reversal can be performed using different techniques: by hand sewing or via a mechanical stapler device. Both techniques were shown to be feasible and safe, although in the meta-analysis by Madani et al., a larger number of small bowel obstructions following the hand sewing technique was observed [3,4]. The option to use different techniques during this type of surgery makes ileostomy reversal a very practical procedure to use while training surgical residents in colorectal surgery. Moreover, the healing of small bowel anastomoses was shown to be better than the healing of large bowel anastomoses, perhaps due to the increased collagenase activity in the colon. This enzyme activity, especially in the first few days, causes a transient decrease in anastomotic strength and could be responsible for the higher anastomotic leakage rate of colonic anastomoses [5]. Nevertheless, ileostomy reversal also poses the risk of perioperative complications. The majority of complications reported are minor complications, such as surgical site infections or diarrhea [6]. However, severe complications, like anastomotic leakage, can also occur, particularly in diabetic patients or if complications arise during initial surgery [7]. The safety of other basic procedures performed by residents under supervision, namely laparoscopic cholecystectomy, has been demonstrated in several studies around the world and also in Poland. Prolonged operation times were understandable during the learning process and generally well accepted by senior colleagues [8,9]. Large meta-analyses of surgeries performed by supervised residents have shown lower mortality rates and a shorter LOS. On the other hand, the heterogeneity of the analyzed patients was very high, and the authors underline that cases for residents should be carefully selected to prevent postoperative morbidity and mortality [10]. The aim of our study is to assess the safety of ileostomy reversal performed by surgical residents.

## 2. Materials and Methods

Between October 2022 and December 2023, a multicenter prospective cohort study titled LILEO (LIquidation of ILEOstomy) was performed in Polish surgical departments. The study analyzed short-term outcomes of the ileostomy reversal procedure with a special focus on perioperative care and postoperative complications. The study was approved by the Bioethical Committee of the Andrzej Frycz-Modrzewski University in Cracow, Poland (KBKA/55/2/2022). Cooperating surgical centers received a detailed study protocol that consisted of inclusion and exclusion criteria, and precise instructions on prospective database collection. In addition, a unified patient information and consent form was distributed to the involved surgical centers. Full data were received via a specially designed password-protected database from 20 surgical departments. Patients were divided into 2 groups: one group was composed of patients operated by specialist surgeons and the other group was made up of patients operated by residents.

The inclusion criteria were as follows: patients who underwent ileostomy, were 18 years old or older, and gave informed consent to partake in the study. Patients who underwent ileostomy reversal as part of a bigger surgical procedure due to colorectal metastases, such as the resection of an obstructing sigmoid tumor or a hemihepatectomy, were excluded from the study.

The primary goals of the study were to determine the postoperative complication rate, LOS, and the 30-day reoperation rate. The secondary goals included assessment of perioperative complications according to their severity measured by the Clavien–Dindo scale and analysis of loop ileostomy reversal postoperative outcomes.

Statistical analysis was carried out using Statsoft STATISTICA v.13 (StatSoft Inc., Tulsa, OK, USA). Numerical variables are presented as the mean and/or standard deviation (SD), with median and interquartile range (IQR) used when applicable. Categorical variables are expressed as percentages. Pearson’s chi-square test of independence was employed to assess the relationship between each variable and the outcome. The Shapiro–Wilk test was used to verify normality for normally distributed data. For groups of quantitative variables that did not follow a normal distribution, comparisons were made using the Kruskal–Wallis test. A *p*-value of less than 0.05 was considered statistically significant.

## 3. Results

Overall, there were 199 patients with full data. Specialists operated in 139 cases assigned to Group 1, while residents operated in 60 cases assigned to Group 2. There were more females in Group 1 than in Group 2 (40.3% versus 20%, *p* < 0.05). Other demographic parameters, such as median age, median body mass index (BMI), and the American Society of Anesthesiologists (ASA) scale did not differ in a statistically significant way. The analysis of comorbidities, namely ischemic heart disease, hypertension, diabetes mellitus, and inflammatory bowel disease of both groups also did not differ significantly. The demographic and comorbidity analysis is presented below in Table 1.

The majority of ileostomies were created during primary surgeries conducted by specialist surgeons in both groups (93.5% versus 96.7%, *p* = 0.373). The median time from ileostomy creation was similar in both groups: 6 months (4–9) in Group 1, and in Group 2, it was also 6 months (4–8), *p* = 0,74. In both groups, there were no significant differences in terms of the technique used during ileostomy reversal; hand-sewn anastomoses and stapler anastomoses were similarly performed by both groups (*p* = 0.414). The median operation time was 75 min (range 60–115) for specialists, and 85 min (range 65–110) for residents, with no statistically significant difference found (*p* = 0.488). In the analysis of wound closure techniques, specialists used single sutures (67.6%), purse-string sutures (21.6%), negative pressure-assisted wound closure (1.4%), and sometimes atypical methods, such as continuous intracutaneous sutures, or delayed sutures (9.4%). In comparison, residents used single sutures (58.3%), purse-string sutures (26.7%), and negative pressure-assisted wound closure (15%). The difference between wound closure techniques used by both groups was statistically significant (*p* < 0.05). A detailed analysis of the surgical/technical aspects of the performed ileostomies is presented below in Table 2.

The median LOS was shorter in the resident group (5.5 days versus 6 days, *p* < 0.05). The number of patients with complications did not differ significantly between the groups, with a 33.1% complication rate in the specialist group and a 21.7% rate in the resident group (*p* = 0.105). The 30-day reoperation rate also did not differ between both groups, with an 8.6% reoperation rate in the specialist group versus a rate of 3.3% in the resident group (*p* = 0.179). The results of postoperative outcomes according to the primary goals of the study are presented in Table 3.

Several patients presented with more than one complication. A further analysis of complication severity showed a higher number of Clavien–Dindo grade 1 and 2 complications in the specialist group (*p* < 0.05), which is presented below in Table 4. Wound infections were the most common postoperative complication, occurring in a total of 33 patients (16.8%), with 22 cases (15.8%) in patients operated on by specialists and 11 cases (18.3%) in patients operated on by residents. Anastomotic leakage was reported in five patients overall (2.5%), including four patients (2.87%) in the specialist group and one patient (1.7%) in the resident group. In addition, it is worth mentioning that Clostridium difficile infection occurred in six patients (4.3%) after ileostomy closure performed by members of the specialist group. There were no differences in severe complications (Clavien–Dindo grades 3a, 3b, 4, and 5) between the two groups.

We also performed a separate analysis solely on loop ileostomy reversals. There were 115 patients operated by specialists and 58 operated by residents. No difference in the LOS was observed: 6 days (5–8) versus 5 days (4–7), respectively; *p* = 0.06. In addition, the overall complication rate did not significantly differ between the two groups, even though the percentage of complications in the specialist group was 30.4%, and in the resident group, it was 18.9% (*p* = 0.107). There was no statistical difference between the two groups in the 30-day reoperation rate, which was 7% in the specialist group and 1.7% in the resident group (*p* = 0.143). The postoperative outcomes for loop ileostomy reversals are presented in Table 5 below.

## 4. Discussion

Educating surgical residents is a challenging task for many reasons. First, training in operating theaters can be stressful for residents. Additionally, the lack of a safe learning environment, unequal learning opportunities, lack of structure and support during residency, and miscommunication between residents and senior colleagues are all considered obstacles in surgical resident education [11,12]. Due to the decreased number of operations conducted during the COVID-19 pandemic, surgical education deteriorated even more because it was transferred online and to training labs [13,14]. Nevertheless, the importance of supervising residents during surgery is a mainstay of becoming an independent surgeon, given that many residents are only prepared theoretically and not practically during their medical education. Kunac et al. reported that the rate of resident autonomy in general surgery cases has decreased by two-thirds in the last 15 years [15]. Thus, more effort should be put into promoting surgical education in operating theaters. Instituting a safety confirmation of surgeries performed by residents is one example.

The majority of ileostomies are still performed by specialist surgeons or surgical consultants. This may be due to the fact that the best outcome for patients undergoing oncological resections, such as low anterior rectal resections with total mesorectal excision and protective loop ileostomies, is often seen when performed by specialized surgeons. As presented by Waters et al., the development of precise training programs for minimally invasive and/or robotic surgery is feasible when residents are performing supervised surgery and leads to safer operating procedures [16]. Also, when a patient is operated on due to inflammatory bowel disease, specialists commonly take over due to expected intraoperative problems that result from inflammation and adhesions that arise after previous surgical interventions [17].

The techniques used during ileostomy reversal in our study are almost evenly distributed between hand-sewn anastomoses and stapler anastomoses. The meta-analysis by Markides et al. demonstrates that stapler anastomoses can be advantageous in reducing operative times and LOS, and that this technique leads to lower rates of postoperative small bowel obstructions [18]. However, a recent randomized control study by Keramati et al., in which handsewn anastomoses are used, demonstrates the opposite: a shorter LOS with better postoperative bowel function in comparison to stapler anastomosis [19]. The aforementioned meta-analysis by Markides et al. and the randomized control study by Keramati et al. show that it is best for residents to learn both techniques and to be able to introduce the technique appropriate for each patient.

In our study, the number of severe complications after all reported ileostomy reversals did not differ between patients operated by residents and those operated by specialist surgeons. Loop ileostomy reversal is associated with a smaller number of perioperative problems and severe postoperative complications [20,21]. Our separate analysis exclusively on loop ileostomy reversal also did not demonstrate differences in the complication rates between the two analyzed groups. A similar safety profile was also seen in other basic operations performed by supervised residents, including Lichtenstein hernia repairs and open appendectomies [22,23]. Given the fact that, unlike in laparoscopic procedures, the overall number of procedures that allow training open techniques among residents is constantly decreasing, ileostomy reversal seems to be the perfect procedure for supervised practice and surgical skill improvement during general surgery training for residents [24,25].

Interestingly, the study by Uecker et al. showed that the LOS was also shorter for many basic surgical procedures, such as cholecystectomy, thyroidectomy, hernia repair, and lower extremity amputation, when residents were involved in the operations. The authors explain that this was the case due to earlier and more frequent visits by resident doctors, and their greater availability and eagerness to participate in discharge preparation and the medication prescription process [26].

Postoperative complications are one of the major factors that result in prolonged hospital stays. An analysis of the possible reasons for postoperative complications by the study group from Barcelona demonstrated that complications were important risk factors during primary surgery when ileostomies were created. Also, patients with chronic kidney disease and with prolonged ileostomy present for more than 10 months can be responsible for postoperative complications, leading to prolonged hospital stays [27]. In practice, when a patient has a severe comorbidity or presents postoperative complications during primary surgery, the residents are less likely to be the operators. In our study, the analyzed comorbidities and the ASA scale were not significantly different between the specialist surgeon and resident groups. However, we had no information about perioperative problems during primary surgery, which can serve as a study limitation. Importantly, there was no significant difference between the two groups in terms of elapsed time after ileostomy creation.

Another thought-provoking aspect affecting postoperative complications is the predominance of female patients in the group operated by specialists. In our study, the overall analysis of ileostomy reversal showed more Clavien–Dindo classification grade 1 and grade 2 complications in the specialist group. In the study by Fleszar et al., it was observed that baseline-adjusted cortisol levels are greater in open surgery performed on females, which can have an impact on postoperative outcomes, especially on surgical site infection levels. Cortisol dynamics correlated with changes in the interleukin levels and also in tumor necrosis factor alpha, which is an interesting finding that requires further investigation [28]. An additional explanation of the aforementioned finding could be that when a specialist is performing the role of supervisor and mentor for the resident, all steps of the surgery are taken slowly and double-checked, which can result in prolonged operating times, but may also increase safety and possibly reduce the number of postoperative complications [29,30].

Our study’s limitations include the variability in surgical techniques and perioperative care practices, stemming from data collected across multiple surgical departments in Poland. Differences in hospital protocols for complication follow-up may account for a higher detection rate of conditions, such as Clostridium difficile diarrhea in patients after ileostomy reversal. Additionally, we did not analyze the surgical training length of residents, which could impact their surgical proficiency and experience. Preoperative preparation for the procedure also varied between the surgical centers involved in this study, which may explain the lower incidence of minor complications, like transient ileus in the postoperative course. Nonetheless, the sample size and prospective character of the analysis allowed us to make conclusions about the safety of ileostomy closure when performed by residents.

## 5. Conclusions

Ileostomy reversal procedures performed by surgical residents under specialist supervision are safe and feasible, with outcomes comparable to those performed by specialist surgeons. These findings show that ileostomy reversal procedures serve as valuable practice tools for developing surgical residents’ skills and do not negatively impact postoperative outcomes.

## Figures and Tables

**Table 1:** Demographic and comorbidity analysis.

	Specialist	Resident	*p* Value
Number of patients, *n* (%)	139	60	-
Females, *n* (%)	56 (40.3%)	12 (20%)	<0.05
Males, *n* (%)	83 (59.7%)	48 (80%)	
Median age, (q1–q3)	65 (56–71)	65.5 (59.5–70)	0.989
Median BMI, kg/m^2^ (q1–q3)	26.5 (23.2–29.4)	29.3 (25.1–32.9)	0.668
ASA 1, *n* (%)	8 (5.8%)	1 (1.6%)	0.348
ASA 2, *n* (%)	82 (58,9%)	40 (66.7%)	
ASA 3, *n* (%)	49 (35.3%)	19 (31.7%)	
Ischemic heart disease, *n* (%)	16 (11.5%)	6 (10%)	0.755
Hypertension, *n* (%)	64 (46%)	28 (46.7%)	0.935
Diabetes, *n* (%)	20 (14.4%)	7 (11.7%)	0.607
History of inflammatory bowel disease			0.668
Crohn’s disease, *n* (%)	5 (3.6%)	1 (1.7%)	
Ulcerative colitis, *n* (%)	4 (2.9%)	1 (1.7%)	

**Table 2:** Surgical aspects analysis.

	Specialists	Residents	*p* Value
Ileostomy created by specialist, *n* (%)	130 (93.5%)	58 (96.7%)	0.373
Ileostomy created by trainee, *n* (%)	9 (6.5%)	2 (3.3%)	
Median time from ileostomy creation, months (IQR)	6 (4–9)	6 (4–8)	0.74
Anastomosis performed during ileostomy reversal:			
Hand-sewn anastomosis, *n* (%)	67 (48.2%)	30 (50%)	0.414
Linear stapler anastomosis, *n* (%)	68 (48.9%)	30 (50%)	
Circular stapler anastomosis, *n* (%)	4 (2.9%)	-	
Median operation time, min (IQR)	75 (60–115)	85 (65–110)	0.488
Wound closure techniques:			
Single sutures wound closure, *n* (%)	94 (67.6%)	35 (58.3%)	<0.05
Purse-string wound closure, *n* (%)	30 (21.6%)	16 (26.7%)	
Negative pressure-assisted wound closure, *n* (%)	2 (1.4%)	9 (15%)	
Other techniques for wound closure, *n* (%)	13 (9.4%)	0	

**Table 3:** Results of postoperative outcomes according to the primary goals of the study.

	Specialist	Resident	*p* Value
Median LOS, IQR (days)	6 (5–9)	5.5 (4–7)	<0.05
Patients with complications, *n* (%)	46 (33.1%)	13 (21.7%)	0.105
30-day reoperation rate	12 (8.6%)	2 (3.3%)	0.179

**Table 4:** Analysis of postoperative complications according to the Clavien–Dindo classification.

	Specialist	Resident	*p* Value
Number of complications in all patients	52 (100%)	15 (100%)	
Clavien–Dindo grade 1, *n* (%)	16 (30.7%)	4 (26.7%)	<0.05
Clavien–Dindo grade 2, *n* (%)	18 (34.6%)	2 (13.3%)	
Clavien–Dindo grade 3a, *n* (%)	2 (3.8%)	7 (46.7%)	
Clavien–Dindo grade 3b, *n* (%)	5 (9.7%)	2 (13.3%)	
Clavien–Dindo grade 4, *n* (%)	9 (17.4%)	-	
Clavien–Dindo grade 5, *n* (%)	2 (3.8%)	-	

**Table 5:** The postoperative outcomes of only loop ileostomy reversal.

	Specialist	Resident	*p* Value
Number of patients, *n* (%)	115	58	
Median LOS, IQR (days)	6 (5–8)	5 (4–7)	0.06
Patients with complications, *n* (%)	35 (30.4%)	11 (18.9%)	0.107
30-day reoperation	8 (7%)	1 (1.7%)	0.143

## Data Availability

Data without personal patient information can be available upon request via email to the main author.
